# Borderline Thrombocytopenia or Mild Idiopathic Thrombocytopenic Purpura?

**DOI:** 10.1371/journal.pmed.0030362

**Published:** 2006-08-29

**Authors:** Jacques Zimmer, François Hentges, Emmanuel Andres

**Affiliations:** Centre de Recherche Public-Santé, Luxembourg; University Hospital of Strasbourg, Strasbourg, France

We read with interest the paper by Stasi et al. [1] about the follow-up of patients with a so-called borderline thrombocytopenia with platelet numbers of 100 × 10^9^/l to 150 × 10^9^/l. Data about the long-term outcome of such patients are indeed not frequent in the literature, as the authors claim, but some information has nevertheless previously been published, as in our retrospective study on adult idiopathic thrombocytopenic purpura (ITP) [2], not cited by Stasi et al. [1].

Our cohort was composed of 201 ITP patients separated by treated and untreated individuals. The latter group was composed of 62 patients (30.8% of the cohort) with a women/men sex ratio of 3.1/1 and a mean age of 39 years.

The vast majority (54 patients, 87.1% of the untreated) were referred to the hospital because of mild isolated thrombocytopenia discovered on routine laboratory examination. These patients were asymptomatic and considered apparently healthy. In eight other cases (12.9%), however, a single, moderate, and spontaneously regressive bleeding episode occurred: purpura (*n* = 4), epistaxis (*n* = 1), gingivorrhagia after tooth extractions (*n* = 1), and meno- and metrorrhagia (*n* = 2) in two women with an intrauterine device and uterine polyps, respectively. The mean platelet count among these patients was 62 × 10^9^/l, compared with 88 × 10^9^/l for the entire untreated group. Antinuclear antibodies were tested in 46 cases (74.2%), with significantly positive values in six patients (13% of the tested individuals). During the follow-up period of 1.9 to 59 months, no further bleeding occurred in the eight patients with initial moderate hemorrhage, although six of them remained thrombocytopenic for more than six months. None of the asymptomatic individuals developed any hemorrhagic symptoms. A chronic ITP (isolated thrombocytopenia of at least six months' duration) was diagnosed in 31 patients (59%) with a mean platelet count of 66 × 10^9^/l. No autoimmune or hematological disease (other than ITP) developed in this group. Twenty-three patients were readdressed to their family physicians for further surveillance and lost to follow-up.

According to current guidelines for the management of adult ITP [3,4], patients with platelet numbers >50 × 10^9^/l are usually not treated. The stable and moderate evolution of the thrombocytopenia in our patients confirms the validity of this approach. The fact that we did not observe the development of other autoimmune diseases in contrast to the small number of such cases noticed by Stasi et al. [1] might be related to the smaller size of our cohort and/or the shorter follow-up period. As these authors define ITP as a persistent platelet count of less than 100 × 10^9^/l and not 150 × 10^9^/l as we did, a direct comparison of the results might be difficult. Whether a patient with platelet counts of less than 150 × 10^9^/l but more than 100 × 10^9^/l is considered to have ITP or borderline thrombocytopenia is an academic question rather than a point of clinical importance, because the medical strategy is exactly the same (surveillance of platelet counts in the absence of any treatment). This holds true for patients with platelets between 50 × 10^9^/l and 100 × 10^9^/l that would be classified as ITP cases according to Stasi et al. [1]. Thus, it is not obvious why an additional clinical entity (borderline thrombocytopenia) should be created in addition to ITP.
